# Increase in Group G Streptococcal Infections in a Community Hospital, New York, USA

**DOI:** 10.3201/eid1506.080666

**Published:** 2009-06

**Authors:** San S. Wong, Yu S. Lin, Liby Mathew, Latha Rajagopal, Douglas Sepkowitz

**Affiliations:** Long Island College Hospital, Brooklyn, New York, USA

**Keywords:** Streptococci, group G streptococci, hospital, New York, USA, letter

**To the Editor:** Identified in 1935 by Lancefield and Hare, group G streptococci (GGS) are part of the normal flora of the pharynx, gastrointestinal tract, genital tract, and skin (*1*–*3*). However, previous case reports have indicated that GGS also could cause complicated infections, including cellulitis, osteomyelitis, septic arthritis, meningitis, endocarditis, and bacteremia (*3*–*6*). Since the mid-1980s, several studies worldwide have reported an increasing incidence of GGS bacteremia (*1*,*5*–*8*), but no recent study has been conducted in the United States to determine the incidence of overall GGS infection.

We noticed that an increasing number of patients with GGS have been admitted to Long Island College Hospital in Brooklyn, New York, USA, during the past few years. To better understand the trend of GGS infection in our institution, we retrospectively reviewed charts of patients admitted from January 2003 through December 2007 who had microbiologically proven GGS infection. Inclusion criteria were clinically and microbiologically documented GGS infection in patients who received appropriate antimicrobial drugs and were >18 years of age. Lancefield GGS were identified in the laboratory by latex agglutination test; resistance profiles were not done for GGS.

A total of 73 persons with GGS were admitted to the hospital during the 5-year study period; the number of patients admitted increased yearly (Figure). Mean age of patients was 53 years; most (77%) were <65 years of age; 52% were women, and most (61%) patients were African American. Thirty (41%) patients had polymicrobial infections; other identified organisms included methicillin-susceptible *Staphylococcus aureus* (8 [11%]), methicillin-resistant *S. aureus* (MRSA) (9 [12%]), and gram-negative or anaerobic organisms (13 [18%]).

**Figure:**
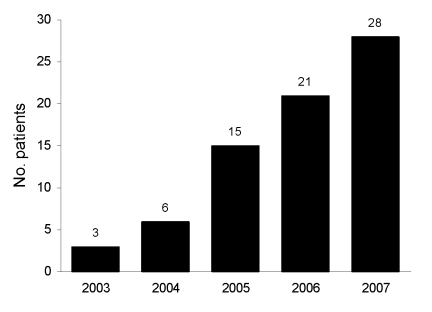
Annual number of patients with group G streptococcal infections admitted to Long Island College Hospital, Brooklyn, New York, USA, 2003–2007.

The spectrum of GGS infections ranged from mild skin and soft tissue infection (34 [46%]) to invasive diseases, including urogenital infection (7 [10%]); lower respiratory tract infection (7 [10%]); pharyngitis (6 [8%]); endocarditis and catheter infection (5 [7%]); and others (14 [19%]), such as peritonitis, pelvic abscess, rectal abscess, and septic arthritis. Four of the 6 persons with pharyngitis were assumed to be colonized with the organism. Eight (24%) of 34 skin and soft tissue infections were associated with bacteremia, 5 (15%) with osteomyelitis, and 20 (59%) with polymicrobial infections. Six persons with lower respiratory tract infections and 1 each with endocarditis, genital tract infection, pelvic abscess, and dental abscess also had polymicrobial infections.

Eighteen persons had bacteremia, the trend of which also increased yearly. Of these, 8 had skin and soft tissue infections, 4 had endocarditis, 2 had urinary tract infections, 1 had possible spontaneous bacteria peritonitis, and 1 had hemodialysis catheter infection; 2 were of unknown source. Of the patients with endocarditis, 2 had vegetations on the native valves, 1 had a pacemaker infection, and 1 had prosthetic valve vegetation. One case of native valve endocarditis occurred in a tricuspid valve in an injection drug user. Another case occurred in a patient in which an epidural abscess was associated with an aortic valve vegetation.

Most of the patients had underlying medical conditions; 34% had diabetes mellitus. In contrast to previous reports, which stated that malignancy was the most common underlying disease (*2*,*3*), only 7 (10%) of the patients in our study group had underlying malignancy, of whom 4 had active malignancy and the rest had had previous malignancy. Nine patients with a history of injection drug use and 5 with HIV infection were identified; the patient with bacteremia secondary to hemodialysis catheter infection had a history of both HIV and intravenous drug use.

Three (4%) patients died; their deaths were unlikely to be attributable to GGS because all were elderly (78–92 years) and had underlying coexisting conditions and co-infections. All 4 persons with endocarditis and the patient with the catheter infection survived. Five patients who were co-infected with MRSA were treated with vancomycin or daptomycin; the remainder were treated with β-lactam antimicrobial drugs and had the sources of infection (catheter or pacemaker) removed. When infections caused by gram-negative or anaerobe organisms were identified, they were also treated with appropriate antimicrobial drugs. The overall average length of stay for all patients with GGS was 9.4 days, with longer stays for those with underlying diabetes mellitus (14.6 days) than for those without diabetes (6.7 days).

GGS was an important etiologic agent for a wide spectrum of infections. Its impressive increase in our institution during the past 5 years raises concerns because other types of β-hemolytic streptococcal infection have increased recently. Group A and B (*9*,*10*) increased substantially during the 1980s. A multicenter analysis may confirm GGS as an emerging human pathogen and may help us better understand the reason for this increase.
